# Unusual Spin Polarization in the Chirality-Induced Spin Selectivity

**DOI:** 10.1021/acsnano.2c07088

**Published:** 2022-10-25

**Authors:** Yotam Wolf, Yizhou Liu, Jiewen Xiao, Noejung Park, Binghai Yan

**Affiliations:** † Department of Condensed Matter Physics, Weizmann Institute of Science, Rehovot7610001, Israel; ‡ Department of Physics, Ulsan National Institute of Science and Technology (UNIST), Ulsan, 44919, Republic of Korea

**Keywords:** chirality, spin polarization, molecular spintronics, spin flip, scattering matrix, quantum transport

## Abstract

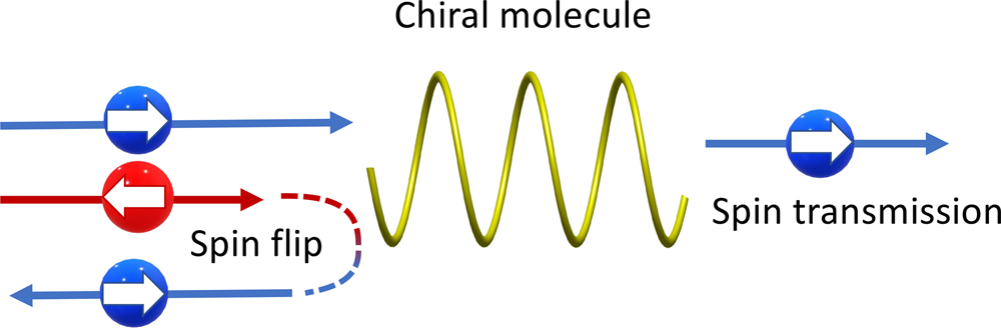

Chirality-induced spin selectivity (CISS) refers to the fact that electrons get spin polarized after passing through chiral molecules in a nanoscale transport device or in photoemission experiments. In CISS, chiral molecules are commonly believed to be a spin filter through which one favored spin transmits and the opposite spin gets reflected; that is, transmitted and reflected electrons exhibit opposite spin polarization. In this work, we point out that such a spin filter scenario contradicts the principle that equilibrium spin current must vanish. Instead, we find that both transmitted and reflected electrons present the same type of spin polarization, which is actually ubiquitous for a two-terminal device. More accurately, chiral molecules play the role of a spin polarizer rather than a spin filter. The direction of spin polarization is determined by the molecule chirality and the electron incident direction. And the magnitude of spin polarization relies on local spin–orbit coupling in the device. Our work brings a deeper understanding on CISS and interprets recent experiments, for example, the CISS-driven anomalous Hall effect.

## Introduction

Chirality-induced spin selectivity (CISS) is a fascinating effect where electrons get spin polarized after propagating through chiral organic molecules like DNA^1−4^ and inorganic materials such as oxides^5−7^ and perovskites.^8−11^ CISS reveals an intriguing relation between the structural chirality and the electron spin or orbital^12,13^ and is promising to design spintronic devices using chiral molecules,^14^ realize enantiomer separation,^15^ and study the spin-selective biological process.^3,4^ Despite the debate on the microscopic mechanisms (see ref (16) and references therein), the chiral molecule is widely regarded as a spin filter.^17−20^

**Figure 1:**
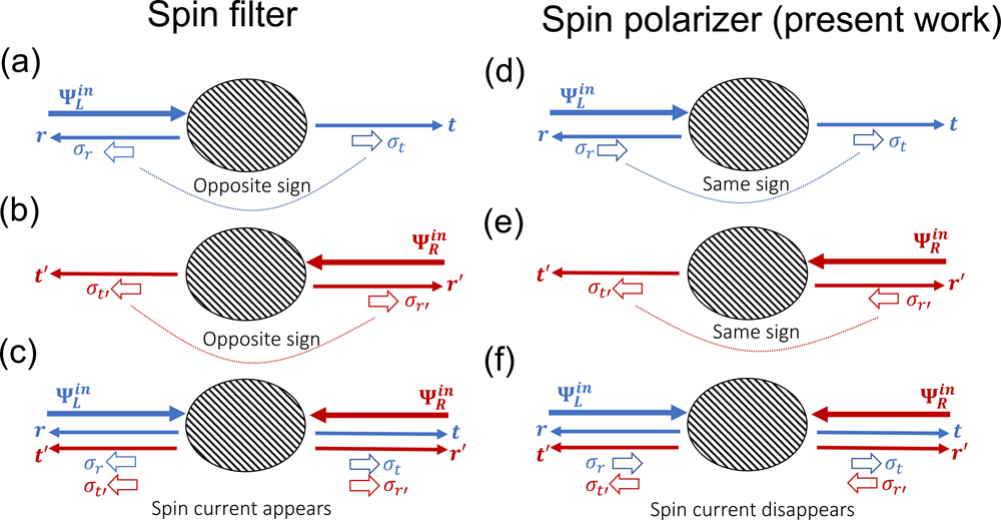
Schematics of transmission and reflection in a two-terminal device. The incident wave from the left (right), , gets scattered with transmission rate *t* (*t*′) and reflection rate *r* (*r*′). The transmission and reflection exhibit spin conductance, σ_*t*_ (σ_*t*′_) and σ_*r*_ (σ_*r*′_), respectively. The spin filter requires that σ_*t*_ (σ_*t*′_) and σ_*r*_ (σ_*r*′_) have opposite signs in (a) [(b)]. In (c) electrons come from the left  and right  equally, which represents the equilibrium state. Then, the left (*r*, *t*′) and right (*t*, *r*′) scattered waves carry opposite spins. Therefore, the equilibrium spin current emerges in (c), which is unphysical. In contrast, the spin polarizer leads to the same sign in σ_*t*_ (σ_*t*′_) and σ_*r*_ (σ_*r*′_) in (d) [(e)]. Then, the equilibrium spin current can be avoided in (f) if eqs 1 and 2 hold.

The CISS spin filter represents that the chiral molecule exhibits a selected transmission rate in one spin channel compared to the opposite spin, in which the preferred spin depends on the chirality. The spin filter was presumed to induce opposite spin polarization in transmitted and reflected electrons (see Figure 1a). This scenario was further generalized to argue that a chiral molecule exhibits transient, opposite spin polarization at two ends in the charge displacement process (see ref (4) for review). In the literature, the spin filter is frequently adopted to rationalize CISS experiments such as the magnetoresistance,^21−33^ anomalous Hall effect (AHE),^34,35^ and the selected chiral adsorption.^15,36^ Such a spin filter scenario is plausibly based on an elusive argument that the total spin density remains zero to preserve the net spin polarization. However, it is established that spin polarization is not necessarily conserved in transport by earlier studies on spintronics, for example, the Rasba–Edelstein effect, where the current leads to net spin polarization in a nonmagnetic material.^37,38^ Therefore, this well-accepted model deserves more examination.

In this work, we point out that the present spin filter picture is incompatible with the prohibition of equilibrium spin current or the general time-reversal symmetry analysis. We prove that both transmitted and reflected electrons present the same type of spin polarization in a two-terminal CISS device based on the unitarity of the scattering matrix. Chiral molecules play the role of a spin polarizer (Figure 1d) rather than a spin filter, because they polarize all scattered (transmitted and reflected) electrons in the same direction, which relies on the molecule chirality and electron incident direction. The spin polarizer picture provides further understandings on CISS, especially on the CISS-driven anomalous Hall effect and the transient spin polarization of chiral molecules in dynamical chemical processes. The scope of the preset work is to understand the current-induced spin polarization from a nonmagnetic CISS device. We ignore the case involving magnetic electrodes in which CISS-induced magnetoresistance is commonly measured and exhibits essential features beyond the spin polarization.^39−41^

## Results and Discussion

### Prohibition of Equilibrium Spin Current

We will discuss a generic two-terminal device with nonmagnetic electrodes. It is established that the equilibrium spin current is strictly forbidden between two terminals because of the time reversal symmetry.^42^ In the quantum scattering problem, electrons come in from the left or right to the center region and are transmitted (*t* from the left, *t*′ from the right) or reflected (*r* from the left, *r*′ from the right), as shown in Figure 1. We denote the spin conductance of a scattering state as σ_*i*_ = *i*_*↑↑*_ – *i*_*↓↓*_ where *i* = *t*, *t*′, *r*, *r*′ is a density matrix. We note that the spin conductance is different from the ratio of spin polarization.

As shown in Figure 1, we denote by “spin filter” the scenario that transmission and reflection have opposite spin polarization and by “spin polarizer” the scenario that they have the same sign. In a CISS device, the 180° rotation of the molecule gives rise to the same sign of spin polarization in transmission because the rotation does not change the chirality. For given chirality, the polarization direction of σ_*t*_ is locked to the transmission direction in a parallel or antiparallel way. Thus, σ_*t*_ is expected to be opposite to σ_*t*′_ in sign because of opposite transmission directions. In equilibrium, electrons are equally incident from and scattered to the left and right. The spin filter presents an equilibrium spin current because left-polarized (σ_*r*_, σ_*t*′_) and right-polarized (σ_*t*_, σ_*r*′_) spins simultaneously move to the left and right, respectively, in Figure 1c.

In contrast, the spin polarizer can avoid the spin current at equilibrium as long as the spin conductance satisfies the condition (Figure 1f)
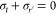
1
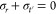
2We will prove that this condition is guaranteed by the unitary property of the scattering matrix in the following.

### Scattering Matrix and Spin Polarization

In the two-terminal device, we define the scattering matrix in the usual notation, as the matrix relating the incoming waves,  and , to the outgoing waves,  and :
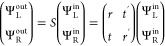
3The unitary property  of the *S*-matrix leads to
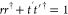
4
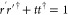
5

 and  are incoming wave functions from the left and right leads, respectively. By assuming *N* independent channels in each lead,  have 2*N* dimensions because of the spin degeneracy. The density matrix ρ_*i*_ of the scattered wave (*i*) can be expressed as
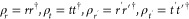
6

It is convenient to calculate the spin conductance of a specific state (*i*) using the corresponding density matrix (ρ_*i*_). Denoting the transport axis as the *z* direction, the spin conductance is
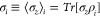
7Combining eqs 4,5,6, and 7, we can derive eqs 1 and 2, i.e., the condition to avoid equilibrium spin current. We must stress that eqs 1 and 2 are generic for any two-terminal devices with nonmagnetic leads, as long as the unitarity of the *S*-matrix holds.

For a symmetric CISS device, the 2-fold rotation symmetry requires σ_*t*_ = −σ_*t*′_. From eqs 1 and 2 we obtain
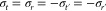
8A similar relation to eq 8 was also derived based on the Onsager’s reciprocal relation in refs (40), (43), and via the unitarity of the scattering matrix in ref (44), all with the assumption of a 2-fold rotation symmetry. In a general CISS device beyond the 2-fold symmetry constraint, we relax the above conditions to σ_*t*_ and σ_*t*′_ having opposite signs. From eqs 1 and 2, we obtain that transmission and reflection have the same sign in spin conductance (thus also the same sign of spin polarization),
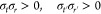
9These results support the spin polarizer scenario rather than the spin filter.

The spin polarizer mechanism requires a spin flipping process upon reflection (also discussed in refs (40) and (43)) such that transmitted and reflected electrons have the same sign of spin polarization. This spin-flipping mechanism can also be understood from a simple time-reversal symmetry argument as illustrated in Supplementary Figure S1. The spin polarizer picture remains unchanged under time-reversal operation of the reflection process while the spin filter scenario violates time-reversal symmetry.

More generally, the above results hold for the conductance of any operator  that satisfies . Detailed derivations based on the outcome of the time-reversal symmetry are presented in Supplementary Section I. For example, the orbital angular momentum operator *L* is such an operator, and angular momentum polarization of transmission and reflection also exhibit the same sign. In summary, the chiral molecule serves as a polarizer, rather than a filter, for spins or orbitals^12^ of conducting electrons.

The above discussion holds for the coherent transport, in which the unitarity condition of eqs 4 and 5 holds. For a dissipating system, we can include dissipation by modifying the unitarity condition , where δ is a uniform dissipation of all the modes and ϵ*A* is a selective dissipation term for the different modes. The matrix *A* is normalized such that its eigenvalues are no larger than 1, and ϵ controls the magnitude of selective dissipation. Following the same process as before, by using eqs 6 and 7 and taking the trace on the modified unitary conditions, we get
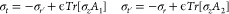
10where *A*_1_ is the left upper block of *A* and *A*_2_ is the right lower block. We see that the uniform dissipation δ does not play a role, only the part that creates selectivity in dissipation of the different modes, and that is bound by ϵ*Tr*[σ_*z*_*A*_1_] and ϵ*Tr*[σ_*z*_*A*_2_]. The trace of σ_*z*_*A*_*i*_ is no larger than *Tr*[|*A*_*i*_|] ≤ 2*N*. As there are 2*N* independent modes in each lead, we normalize the spin conductance by 2*N* in order to have −1 ≤ σ ≤ 1. Thus, we get
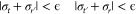
11Assuming σ_*t*_ = −σ_*t*′_, we get
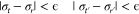
12From this, we see that if the spin conductance is larger than the magnitude of the selective dissipation term, then the conductance of transmission and reflection spin have the same sign. The intuitive explanation to why selective dissipation of modes alters the spin conductance induced by the chirality of the molecule is that selective dissipation is an orthogonal mechanism that can theoretically polarize spin as well; for example, selective dissipation of spin-down states in an unpolarized current leaves the current spin polarized in the up direction.

### Quantum Transport Calculations

**Figure 2:**
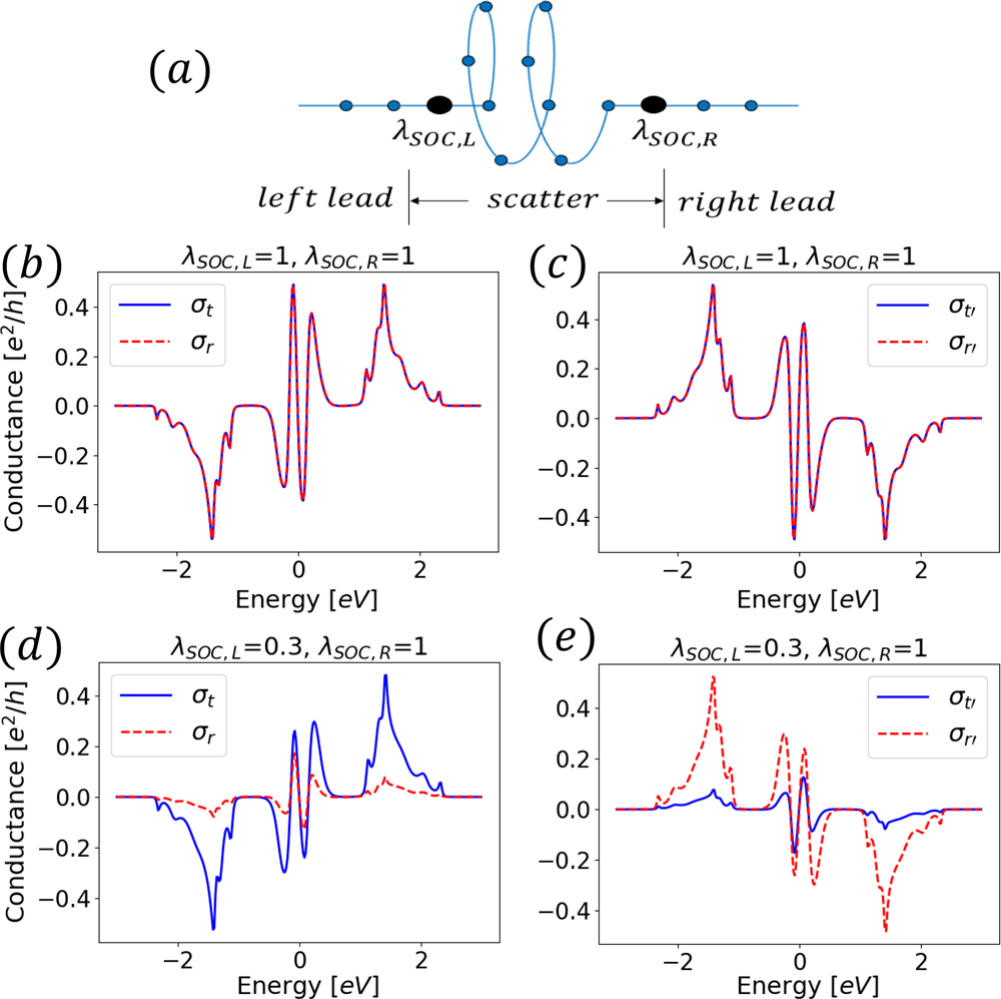
(a) Generic structure of the CISS device, including the leads and interface. (b, c) Calculated spin conductance (σ_*i*_ = *i*_*↑↑*_ – *i*_*↓↓*_, where *i* is a scattering state) of *t*, *r*, *t*′, and *r*′ in a *C*_2_ symmetric system at different Fermi energies. (d, e) The spin conductance in a *C*_2_-symmetry-breaking system by differentiating λ_SOC,L_ and λ_SOC,R_. λ_SOC,L/R_ represents the value of spin–orbit coupling at the left/right interface.

To examine the above analytic results, we performed Landauer–Bütikker quantum transport calculations on two-terminal CISS devices. The device is composed of a helical chain sandwiched between half-infinite linear chains, as illustrated in Figure 2a. Each site has p_*x*,*y*,*z*_ orbitals and two spins. Between sites, we set Slater–Koster-type^45^ hopping parameters to nearest neighbors. Because the nearest-neighbor *p*_*x*,*y*,*z*_ hopping parameters depend on the relative atomic positions in a chiral chain, these hopping parameters manifest the chirality by picking up opposite phases for opposite chiralities (see more details in Supplementary Section II). We only set finite spin–orbit coupling (SOC, λ_SOC_) at two interface sites, to represent that electrodes exhibit dominantly larger λ_SOC_ than the chiral molecule in a CISS device. However, our main conclusions will be independent of whether λ_SOC_ comes from the molecule or electrodes. The spin polarization in both terminals is well-defined in the calculations because there, we set the SOC to zero. All the conductance calculations were performed with Kwant.^46^ Parameters of the model and band structure of leads and chiral chain can be found in Supplementary Section II.

Figure 2b,c show the case of a symmetric device. The transmission and reflection are calculated for different Fermi energies, which fully agree with eq 8. After violating the *C*_2_ symmetry by setting λ_SOC_, which differ between the two interfaces (Figure 2d,e), σ_*t*_ and σ_*r*_ (σ_*t*′_ and σ_*r*′_) are not equal in value, but eqs 1, 2, and 9 are always valid.

Up to this point in the subsection, we presented quantum transport calculations on a specific model to demonstrate the validity of the spin polarizer mechanism. We should point out that the spin polarizer conclusion comes from the unitarity of the scattering matrix and is independent from any details (orbital, spin, or SOC) of the model.

**Figure 3:**
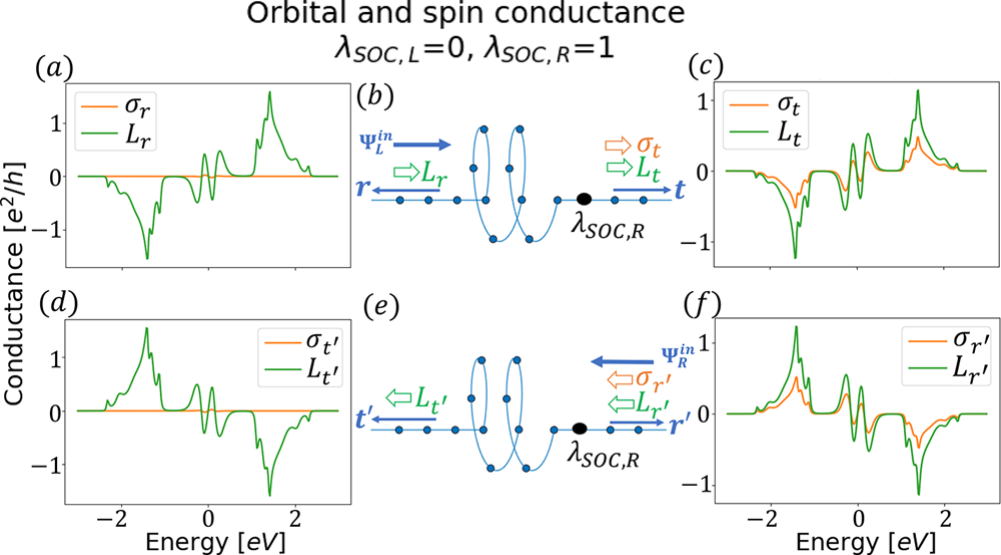
Orbital and spin conductance in a system where *C*_2_ symmetry is broken by having SOC only on the right side. (a–c) Orbital and spin conductance of *r* and *t*: Current coming from the left gets orbital polarized along the chain; then the orbital current is converted to spin current by the SOC atom. (d–f) Orbital and spin conductance of *r*′ and *t*′: Current coming from the right hits the SOC atom before getting orbital polarized, so it passes unaltered into the chain, where it gets orbital polarized, after which it passes to the left lead only orbital polarized.

Figure 2d,e already demonstrate sensitive dependence of the spin conductance on SOC. Furthermore, we set an extreme example with λ_SOC,L_ = 0 and λ_SOC,L_ = 1 in Figure 3 and calculate both the spin and orbital (*L*) conductance here. One striking feature is that σ_*r*_ and σ_*t*′_ are diminished in amplitude, while σ_*r*′_ and σ_*t*_ are still significant. Because the orbital is less sensitive to λ_SOC_, the orbital conductance *L*_*r*,*t*,*r*′,*t*′_ exhibits a large amplitude and satisfies
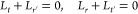
13
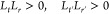
14One intuitive picture is that electrons get orbital polarized in the chiral molecule and the interface SOC converts the orbital polarization to spin polarization. For example, λ_SOC,R_ converts large *L*_*t*_ (*L*_*r*′_) to σ_*t*_ (σ_*r*′_), while there is no λ_SOC,L_ to induce σ_*r*_ (σ_*t*′_) from *L*_*r*_ (*L*_*t*′_). In addition, the tiny amplitude of σ_*r*_ (σ_*t*′_) at some energies is induced by the weak interface orbital–spin conversion due to λ_SOC,R_.

In the orbital–spin conversion, we observe a counterintuitive effect that the transmitted/reflected spin conductance remains the same when reversing the sign of λ_SOC_ (see Supplementary Figure S3). It becomes rational when considering that SOC connects states with the same total angular momentum *J*_*z*_ = *L*_*z*_ ± *S*_*z*_ as a scattering potential. When treating SOC perturbatively, no matter the sign of the SOC, the allowed transitions between the chiral chain and electrode are dictated by nonzero matrix elements of the SOC Hamiltonian (“selection rules”). Suppose the orbital polarization in the chiral chain will be in favor of *L*_*z*_ = +1 and equal probability for . Direct calculation of these matrix elements shows that the allowed transitions are
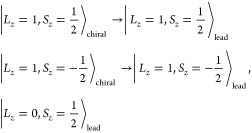
We can see that these selection rules allow the positive orbital polarization to convert spin states, from down to up, by trading angular momentum between orbital and spin, while an up–down conversion of spins is not allowed. Thus, the positive orbital polarization leads to the positive spin polarization when scattered by the SOC site. Similarly, a negative orbital polarization generates negative spin polarization. The orbital and spin relation is shown by the same sign between σ_*t*_ and *L*_*t*_ (also σ_*r*′_ and *L*_*r*′_) in Figure 3.

### Discussion on Experiments

**Figure 4:**
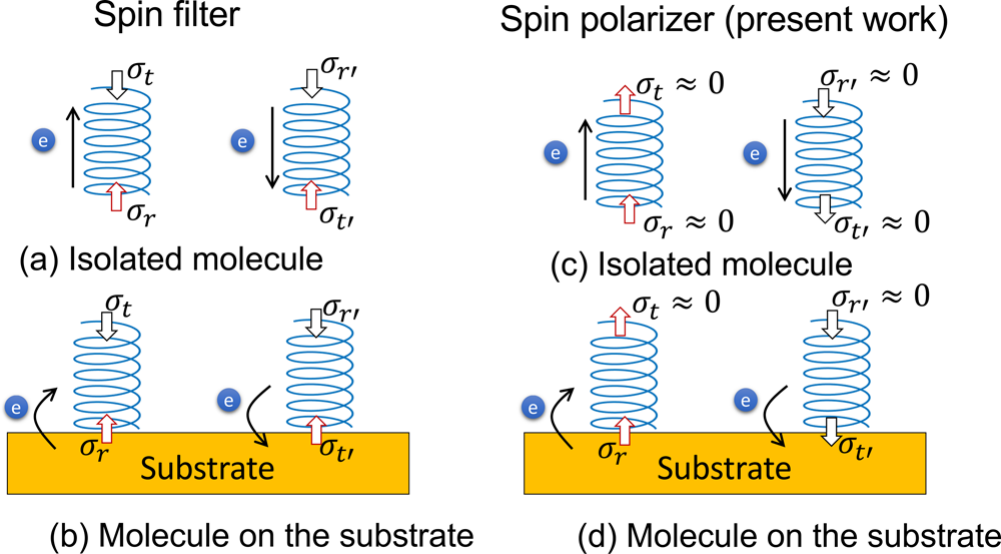
Transient spin polarization in (a) and (c) of the isolated chiral molecule and (b) and (d) a chiral molecule on the surface. The instantaneous charge redistribution is indicated by the black arrow. The spin conductance σ_*i*_ represents the transient polarization and is defined in the same way as in Figure 1.

As discussed above, the direction of the spin polarization is determined by the current direction and molecule chirality, while the magnitude of spin polarization depends on the local SOC. This observation provides insights on the CISS-induced transient spin polarization of chiral molecules. In chemical reactions or surface adsorption, it is commonly argued in the literature^15,36,47−50^ that the instantaneous charge displacement leads to opposite spin polarization at both ends of a chiral molecule (as illustrated in Figure 4a,b) by assuming the spin filter scenario. Following the picture of a spin polarizer, we expect that both ends exhibit the same sign of spin polarization (see Figure 4c). If the chiral molecule is isolated far from a strong SOC region (e.g., a substrate or electrode), the magnitude of spin polarization may be negligible because of weak SOC. If the molecule is close to a heavy metal surface (Figure 4d), the interface region may develop substantial spin polarization. Additionally, such transient spin polarization may vanish soon after the spin lifetime when the system approaches equilibrium.

It is noteworthy that the spin polarizer mechanism gets more rational when one considers the previous AHE experiments.^34,35^ When the top gate ejects/extracts electrons through a layer of chiral molecules into/out of doped-GaAs or GaN, the magnetization was induced in the doped semiconducting layer and monitored by the AHE. Switching the gate voltage was found to reverse the sign of induced magnetization in the semiconductor. The spin polarizer naturally indicates opposite spin polarizations in the semiconductor after reversing the tunneling direction for the given chirality (Figure 1d,e).

This is consistent with the experimental observation that the AHE changes sign after reversing the gate voltage over the chiral molecule layer.^34,35^ In contrast, the spin filter scenario would indicate no sign change in the semiconductor side (Figure 1a,b) after switching the gate voltage.

**Figure 5:**
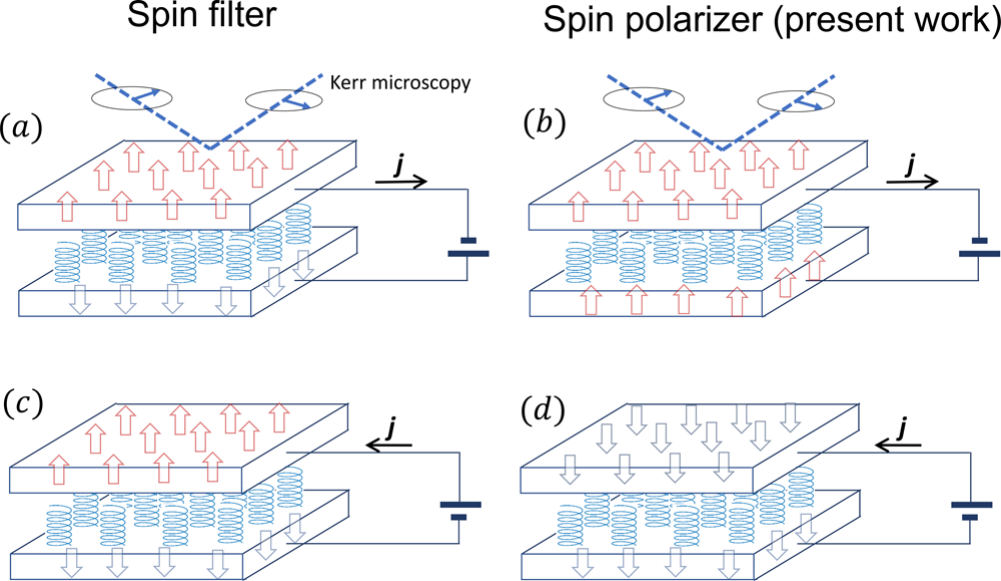
Schematics of a two-terminal device where two nonmagnetic electrodes sandwich a chiral molecule thin film. The current-induced magnetization (large red and blue arrows) on the top electrode can be measured with scanning SQUID or Kerr microscopy. (a and c) Spin polarization in a spin filter scenario where the current-induced magnetization remains the same after reversing the current (*j*). (b and d) Spin polarization in a spin polarizer scenario where the current-induced magnetization switches sign after reversing the current.

We propose that the induced spin polarization in electrodes can also be detected by spectroscopies that are sensitive to surface magnetization, e.g., the magneto-optical Kerr effect^51^ or the scanning SQUID.^52,53^Figure 5 depicts the schematic for a two-terminal CISS device with nonmagnetic electrodes sandwiching a chiral film. As current is driven through the system, both electrodes get spin polarized. The induced magnetization on the top electrode can be detected by a Kerr microscope or scanning SQUID. The magnetization detected this way would remain with the same sign for both current flow directions in the spin filter scenario but would change sign according to the spin polarizer mechanism, unambiguously discriminating the two mechanisms. In this experiment, the amplitude of the magnetization will be proportional to the current density and also the magnitude of SOC in the top electrode.

## Conclusions

In summary, we proposed that the spin polarizer model is more rational than the spin filter for the chiral molecule in the CISS device. Here, the transmitted and reflected electrons exhibit the same sign in spin polarization as a leading order effect. This scenario provides a deeper understanding on the CISS-driven spin polarization and alternative explanations on the induced AHE and transient spin polarization. The spin polarizer (filter) leads to opposite (same) spin polarization direction in a given electrode when reversing the current direction. Thus, the current-direction-specific spin polarization provides a smoking-gun evidence to verify the spin polarizer effect in experiments.

## Methods

A more general version of the calculation of spin conductance using the scattering matrix can be found in Section I of the Supporting Information, where the relation between transmitted and reflected conductance is derived for a general observable.

All the conductance calculations were performed with Kwant.^46^ Parameters of the model and band structure (Supplementary Figure S2) of leads and chiral chain can be found in Section II of the Supporting Information.

## Supporting Information Available

Section I: Derivation of polarization equality between transmitted and reflected states; Section II: Parameters of model and band structure; Figure S1: Reflection of an electron by a chiral molecule when the electron spin is not favored by CISS; Figure S2: Band structure of the chiral chain and linear chain; Figure S3: Spin polarization for negative SOC on both sides (PDF)
