# Supplementary material for: Unusual Spin Polarization in the Chirality-Induced Spin Selectivity
Source: ACS Nano. 2022 Oct 25;16(11):18601–7. doi: 10.1021/acsnano.2c07088 (PMC9706810; doi:10.1021/acsnano.2c07088)
Supplement: Supplementary file 1 — nn2c07088_si_001.pdf [file nn2c07088_si_001.pdf]

Unusual Spin Polarization in the Chirality Induced Spin Selectivity

Yotam Wolf,[†] Yizhou Liu,[†] Jiewen Xiao,[†] Noejung Park,[‡] and Binghai Yan[*,†]

†*Department of Condensed Matter Physics, Weizmann Institute of Science, Rehovot 7610001, Israel*

‡*Department of Physics, Ulsan National Institute of Science and Technology (UNIST), Ulsan, 44919 Republic of Korea*

E-mail: binghai.yan@weizmann.ac.il

Supplementary information

I Derivation of Polarization Equality Between Transmitted and Reflected States

Here, we shall show that in a two terminal system with no dissipation and leads that respect time reversal symmetry, the polarization of transmitted and reflected currents with respect to a general traceless operator, $\mathcal{O}$, is the same. An incoming or outgoing state from the left or right has $2N$ components, corresponding to N orbitals, each 2-fold degenerate in spin.

In the density matrix formalism, the relation between an incoming state and an outgoing state is given by:

$$\rho_{out} = S\rho_{in}S^\dagger = \begin{pmatrix} r & t' \\ t & r' \end{pmatrix} \begin{pmatrix} \rho_L^{in} & 0 \\ 0 & \rho_R^{in} \end{pmatrix} \begin{pmatrix} r^\dagger & t^\dagger \\ t'^\dagger & r'^\dagger \end{pmatrix} \tag{S1}$$

Carrying out the matrix multiplication yields the outgoing states in the left and right:

$$\begin{pmatrix} \rho_L^{out} & * \\ * & \rho_R^{out} \end{pmatrix} = \begin{pmatrix} r\rho_L^{in}r^\dagger + t'\rho_R^{in}t'^\dagger & * \\ * & r'\rho_R^{in}r'^\dagger + t\rho_L^{in}t^\dagger \end{pmatrix} \tag{S2}$$

When current is incoming only from the left, $\rho_R^{in} = 0_{2N\times 2N}$. In addition, under the assumption of an incoherent sum of modes incoming from the left, $\rho_L^{in} = \mathbb{1}_{2N\times 2N}$. Plugging into the above equation:

$$\rho_L^{out} = rr^\dagger \equiv \rho_r \qquad \rho_R^{out} = tt^\dagger \equiv \rho_t \tag{S3}$$

Similarly, when current is incoming only from the right:

$$\rho_R^{out} = r'r'^\dagger \equiv \rho_{r'} \qquad \rho_L^{out} = t't'^\dagger \equiv \rho_{t'} \tag{S4}$$

From the unitarity condition of the density matrix in eq. (4)

$$\rho_r + \rho_{t'} = \mathbb{1} \tag{S5}$$

Multiplying by $\mathcal{O}$ and taking the trace:

$$Tr[\mathcal{O}\rho_r] + Tr[\mathcal{O}\rho_{t'}] = Tr[\mathcal{O}] \tag{S6}$$

As the trace of $\mathcal{O}$ is zero, and using the generalized form of eq. (7) for expectation value, $Tr[\mathcal{O}\rho_i] = \langle\mathcal{O}\rangle_i$, we obtain:

$$\mathcal{O}_r + \mathcal{O}_{t'} = 0 \tag{S7}$$

Similarly, using the second unitary relation in eq. (5), we obtain:

$$\mathcal{O}_{r'} + \mathcal{O}_t = 0 \tag{S8}$$

When these outcomes are combined with the selectivity inherent to the C_2 symmetric system, $\mathcal{O}_t = -\mathcal{O}_{t'}$, we obtain:

$$\mathcal{O}_r = \mathcal{O}_t = -\mathcal{O}_{t'} = -\mathcal{O}_{r'} \tag{S9}$$

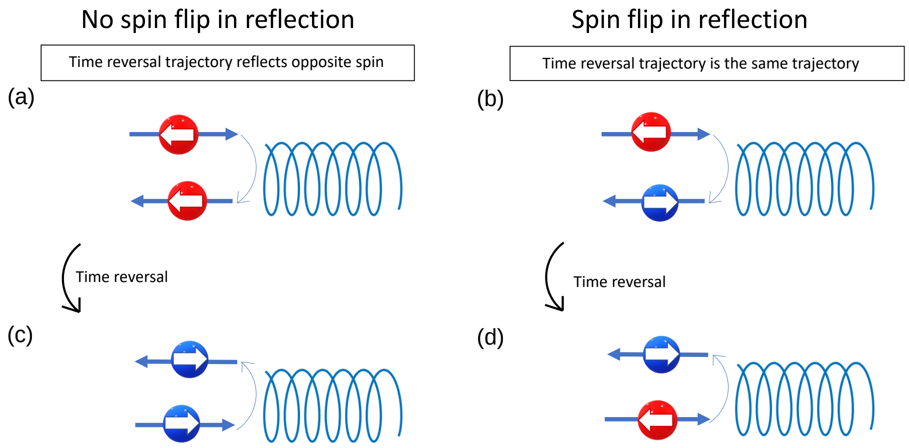

Figure S1: Reflection of an electron by a chiral molecule in front of a non-magnetic lead when the electron spin is not favored by CISS. Since the system is non-magnetic, the time-reversed trajectory (reversing both spin direction and velocity direction) of the electron should preserve the original spin selectivity. (a)&(c) For a spin filter, the reflection involves no spin flip, i.e., the reflected electron has the same spin as the incoming electron. (c) is the time-reversed trajectory of (a). However, (c) indicates the originally favored spin in (a) is reflected (becomes unfavorable). (b)&(d) For a spin polarizer, the reflection involves a spin flip process, i.e., the reflected electron has the opposite spin as the incoming electron. (b) and its time-reversal trajectory (d) are essentially the same process without contradiction, where the same spin gets reflected (remains unfavorable). In summary, the spin selectivity changes as reversing time in (a)&(c) while it remains the same in (b)&(d).

II Parameters of Model and Band Structure

In the model we work with electrons in the basis of the of the 3 p orbitals and the up and down states of spin half: $\{|p_x\rangle, |p_y\rangle, |p_z\rangle\} \otimes \{|\uparrow\rangle\, |\downarrow\rangle\}$. The parameters will all be given in units of eV. In addition, they will be shown as an outer product of 3×3 matrices in the p orbital basis, with 2×2 matrices in the spinor basis.

In the left and right lead, we use the nearest neighbor hopping matrix:

$$t_{lead} = \begin{pmatrix} -2.4 & 0 & 0 \\ 0 & -2.4 & 0 \\ 0 & 0 & 1.6 \end{pmatrix} \otimes \mathbb{1}_{2\times 2} \tag{S10}$$

The on-site matrix term of the left and right lead is set to zero. The band structure of the leads can be seen in Fig. (S2b).

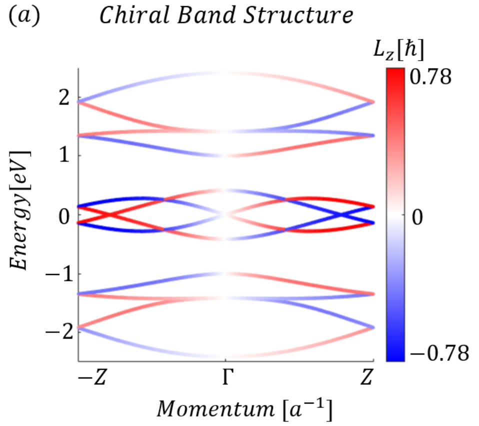

Figure S2: (a) Band structure of chiral molecule in the infinite chain form. The blue and red colors represent the orbital projection. (b) Band structure of the lead.

The interface between the left lead and the chiral molecule contains one atom. The hopping from the lead to this atom is given by the above hopping matrix (S10), and the hopping into the chiral molecule is given by the following hopping matrix:

$$t_{SOC} = \begin{pmatrix} -1.5 & 0 & 0 \\ 0 & -1.5 & 0 \\ 0 & 0 & 1 \end{pmatrix} \otimes \mathbb{1}_{2\times 2} \tag{S11}$$

The onsite term of the atom in the interface is the SOC Hamiltonian:

$$h_{SOC} = \lambda_{SOC,L} \sum_{i=x,y,z} l_i \otimes \sigma_i \tag{S12}$$

Where σ_i are the three Pauli matrices and l_i are the (unitless) angular momentum operators as 3×3 matrices in the p orbital basis. $\lambda_{SOC,L}$ denotes the spin-orbit coupling constant, L indicates that this is the interface with the left lead.

For the interface between the chiral molecule and the right lead, we use the exact same model, we only change the spin-orbit coupling constant $\lambda_{SOC,R}$.

The hopping in the chiral molecule is nearest neighbors and is given by the following matrix:

$$t_{chiral,ij} = \begin{pmatrix} t_{xx} & t_{xy} & t_{xz} \\ t_{yx} & t_{yy} & t_{yz} \\ t_{zx} & t_{zy} & t_{zz} \end{pmatrix} \otimes \mathbb{1}_{2\times2} \tag{S13}$$

$$t_{xx,ij} = t_\pi sin^2\phi_{ij} + cos^2\phi_{ij}(t_\sigma sin^2\theta_{ij} + t_\pi cos^2\theta_{ij})$$
$$t_{yy,ij} = t_\pi cos^2\phi_{ij} + sin^2\phi_{ij}(t_\sigma sin^2\theta_{ij} + t_\pi cos^2\theta_{ij})$$
$$t_{zz,ij} = t_\sigma cos^2\theta_{ij} + t_\pi sin^2\theta_{ij}$$
$$t_{xy,ij} = t_{yx} = sin\phi_{ij} cos\phi_{ij}(t_\sigma sin^2\theta_{ij} - t_\pi cos^2\theta_{ij})$$
$$t_{xz,ij} = t_{zx} = cos\phi_{ij} sin\theta_{ij} cos\theta_{ij}(t_\sigma - t_\pi)$$
$$t_{yz,ij} = t_{zy} = sin\phi_{ij} sin\theta_{ij} cos\theta_{ij}(t_\sigma - t_\pi)$$

where θ_{ij} and ϕ_{ij} are the spherical coordinates of site j with respect to site i. In our model, we use a helix with a C_4-skew symmetry, such that ϕ_{ij} can adopt the angles $\frac{\pi}{8}$, $\frac{\pi}{8}+\frac{\pi}{2}$, $\frac{\pi}{8}+\frac{2\pi}{2}$, $\frac{\pi}{8}+\frac{3\pi}{2}$ and $\theta_{ij} = \pm\frac{\pi}{4}$. In the model, the chiral molecule contains 8 atoms and $t_\pi = -0.5eV$, $t_\sigma = 1.5eV$. The onsite terms in the chiral molecule are set to zero. The sign of ϕ_{ij} indicates the chirality of the chain. If chirality is reversed, we only need flip the sign of ϕ_{ij} in Eq. S13. The band structure of the chiral chain can be seen in Fig. (S2a).

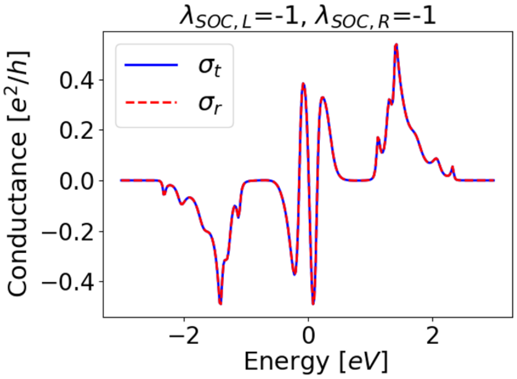

Figure S3: Spin polarization of t and r for negative SOC on both sides. Same sign of polarization as positive SOC.
